# Acceptance and Commitment Training for Ice Hockey Players: A Randomized Controlled Trial

**DOI:** 10.3389/fpsyg.2021.685260

**Published:** 2021-07-22

**Authors:** Tobias Lundgren, Gustaf Reinebo, Markus Jansson Fröjmark, Emil Jäder, Markus Näslund, Per Svartvadet, Ulf Samuelsson, Thomas Parling

**Affiliations:** ^1^Centre for Psychiatry Research, Department of Clinical Neuroscience, Karolinska Institutet, Stockholm Health Care Services, Stockholm County Council, Stockholm, Sweden; ^2^Department of Psychology, Stockholm University, Stockholm, Sweden; ^3^MoDo Hockey, Örnsköldsvik, Sweden

**Keywords:** ACT, ice hockey, randomized controlled trial, performance enhancement, sport psychology

## Abstract

Recent systematic reviews on the topic of mindfulness- and acceptance-based approaches in sport psychology conclude that there is a need for further trials using a more robust research methodology with direct performance as outcome. Acceptance and Commitment Training (ACT) is a contextual behavioral change method that focuses on facilitating psychological processes such as values, committed action, acceptance and mindfulness. In the present study designed as a randomized controlled trial, 34 junior elite ice hockey players were allocated into either an ACT group intervention or a wait list control group. Results showed significant effects on both objective performance outcomes (goals, assists, and taken shots) and blinded coach ratings of players’ performance, focus and commitment to their development in favor of the ACT group. Effects lasted at 3-month follow-up for the coach ratings, but not for the objective performance measures. All ACT trained players recommended ACT to other players and considered the training as important for their development as ice hockey players. The results add to the growing body of evidence on ACT interventions for athletes and its effect on performance. Future studies should investigate the maintenance of effects from the psychological training over time, using robust research methodology and investigate theoretical coherent potential mediating variables.

## Introduction

Psychological skills are an integral part of being an effective athlete in competitive elite sports. Psychological skills training (PST) have been an area of interest for sport practitioners and researchers since the early 1970s. PST has mainly been based on cognitive techniques such as imagery, positive thinking and control of private experiences (Weinberg and Gould, 2019). The research using stringent scientific methods of the effects of these methods are scarce, especially when objective/direct performance is the dependent variable of interest (Gardner and Moore, 2004; Noetel et al., 2019). During the last decade there has been an increased interest in mindfulness and acceptance-based training methods, often derived from Acceptance and Commitment Therapy adapted for athletes (Bühlmayer et al., 2017). Acceptance and mindfulness interventions apply a different focus for the psychological training than traditional PST programs. One central difference between the PST programs and the ACT based interventions is that acceptance and mindfulness interventions aim to train athletes to change the way athletes relate to private experiences (emotions, thoughts, and memories) rather than changing the content of the private experiences (Gardner and Moore, 2004; Birrer and Röthlin, 2017).

Acceptance and Commitment Therapy (ACT) (Hayes et al., 1999) is a contextual behavior therapy model extensively tested to reduce human suffering (Ruiz, 2012; A-Tjak et al., 2015). ACT is theoretically based on behavior analysis and a behavioral theory of language and cognition known as Relational Frame Theory (RFT) (Hayes et al., 2001). The purpose of the ACT approach in clinical settings is to increase psychological flexibility (PF) and in doing so decrease psychological problems and increase quality of life. Mindfulness, values clarification and acceptance are a few central psychological processes that constitute PF. Some of these processes are also at the heart of several other third wave interventions (Hayes et al., 2011). PF is defined as the ability to fully contact the present moment and the thoughts and feelings it contains without needless defense, and depending upon what the situation affords, persisting in or changing behavior in the pursuit of personally meaningful goals and values (Hayes et al., 2006). PF predicts not only mental health and quality of life but also effective workplace performance and work-related well-being (Flaxman and Bond, 2010; Bond et al., 2011). ACT has been evaluated for a range of psychiatric and somatic disorders showing that ACT-interventions reduce symptoms as well as increase life quality often mediated by PF (Powers et al., 2009; Ruiz, 2012; A-Tjak et al., 2015).

When mindfulness- and acceptance-based approaches made their transition into sports about 15 years ago, the adapted methods was heavily influenced by ACT, e.g., the Mindfulness-Acceptance-Commitment approach (MAC) (Gardner and Moore, 2004, 2007). In this section, three recently conducted randomized controlled trials (RCTs) investigating the effects of mindfulness and acceptance-based approaches in sports will be mentioned. Gross et al. (2018) compared traditional PST with MAC in collegiate female athletes. In comparison to PST, MAC showed superior effects on behavioral and emotional measures such as substance abuse, hostility and emotion dysregulation. No objective performance data was included, however, a significant within-group increase in coach rated performance was found for the athletes in the MAC group. Another recent RCT study also compared MAC with PST in athletes from various sports and competitive levels (Josefsson et al., 2019). MAC showed larger effects on mindfulness, emotional regulation and subjective performance compared to PST. Further, both dispositional mindfulness and emotion regulation mediated the effect of MAC on the self-rated performance outcome. This suggests that dispositional mindfulness and emotion regulation may be important target processes of behavioral change in MAC. Thirdly, a three-arm RCT was conducted with athletes randomized to either a four-session workshop of mindfulness training (MT), PST, or a wait-list control. 95 athletes from four sports (curling, tennis, floorball, and badminton) enrolled. MT and PST showed some shared effects such as improving athlete’s emotional skills and attentional control. Effects were also intervention dependent and in line with intervention theory, e.g., MT showed greater reduction in experiential avoidance, which is also more emphasized in MT than in PST thus in line with expectations. Since both MT and PST were beneficial to the athletes, although in different ways, sport psychologists need to take several aspects into account when choosing the most appropriate intervention strategy (Röthlin et al., 2020).

Systematic evaluations of the available evidence suggests that there is some support for the efficacy of acceptance and mindfulness-based interventions on performance surrogates, however the evidence on direct performance is still scarce. Recent systematic reviews stresses the importance for further high-quality trials with a robust methodological approach (Sappington and Longshore, 2015; Bühlmayer et al., 2017; Noetel et al., 2019). Also, to our knowledge, no mindfulness and acceptance-based intervention has been developed specifically to enhance performance for ice hockey players using an RCT design with performance measures as outcome. Hence, a central aim for the present study was to address this absence and investigate an acceptance and commitment training program for ice hockey players using a randomized controlled design and evaluating its effect primarily on direct performance.

The aim of the ACT approach in ice hockey is to develop psychologically flexible ice hockey players (Lundgren et al., 2020). A psychologically flexible player approaches their ice hockey development and game being open and aware of their athletic experience no matter the content of the private events or obstacles they may encounter. Also, the flexible player directs his or her attention toward what is important in given situations and acts in line with their ice hockey related values and goals. *Acceptance* is a central process in ACT, and is defined as the ability to actively and consciously embrace private experiences (thought, feeling, memory, sensation etc.) without trying to escape or avoid them by changing their frequency or form (Hayes et al., 1999). In an ice hockey context acceptance is adapted and defined as an ability to actively embrace ice hockey related emotions, thoughts and memories in such a way that these experiences do not become obstacles to effective performance (Lundgren et al., 2018). Present moment awareness is another central process and is key to the concept of *mindfulness* (Kabat-Zinn, 1994). Mindfulness is defined as the ability to shift the attention to here and now in a flexible and non-judgmental way, contacting both internal and external stimuli with dispassionate observation (Fletcher and Hayes, 2005). In an ice hockey context, mindfulness is defined as the ability to be present and aware on the ice, direct attention effectively in any given situation in order to choose actions effectively during games and practices (Lundgren et al., 2018). *Values* are the verbal descriptions of overarching important goals, also known as verbally established motivation (Plumb et al., 2009). In the ice hockey context, values are defined as the qualitative individual description of how an ice hockey player wants to approach game and training in order to give themselves the best chance to be successful and enjoy their ice hockey (Lundgren et al., 2018).

In a recent controlled group study, ACT was adapted to the ice hockey context to function as a performance enhancement program for elite ice hockey players (Lundgren et al., 2020). The study sample consisted of 21 players in total. 13 players were interested to take part of the ACT training which formed the experimental group. The 8 players that played in the same ice hockey team and declined to take part of the training accepted to be part of the study and therefore served as control group. The purpose of the study was to evaluate the feasibility of the ACT program as well as the effect on ice hockey related PF. Ice hockey related PF was measured using an adapted version of the AAQ-II, the Values, Acceptance and Mindfulness Scale (VAMS). Higher levels of ice hockey related PF measured by the VAMS are significantly associated with better objective performance measures such as assists and team points. Also, higher levels of ice hockey related PF measured with VAMS were significantly associated with higher levels of self-rated quality of life and lower level of mental health distress (Lundgren et al., 2018). The result of the controlled group trial showed that the ACT training program significantly increased ice hockey related PF as compared to the control group with large effect sizes (Cohen’s *d* = 0.86). Also, the participants considered the ACT program both meaningful and helpful, and all participants stated that they would recommend the training program to other ice hockey players. The ACT program proved feasible in the ice hockey context and influenced targeted ACT processes such as values, acceptance and mindfulness significantly. However, there were some limitations to the study. Most importantly, no randomization procedure was used and there was no objective performance data used as outcome. These are some of the main concerns that the current study seeks to address.

The overall aim of the present study is to evaluate the effect of an ACT program on ice hockey player’s direct performance, using a randomized controlled study design. The specific aim of this study is to investigate the effect of an ACT-program for ice-hockey players with the following research questions: (1) To what extent can an ACT based group-intervention increase ice hockey players’ objective performance as measured with goals, assists, shots and plus/minus? (2) To what extent can an ACT based program increase players focus, engagement and performance as measured by blinded coach ratings? (3) To what extent will the players consider the training meaningful and helpful to them as ice hockey players? (4) Will the players recommend the intervention to other ice hockey players? Based on these research questions the following hypotheses are stated:

1. Players in the ACT group will increase their objective performance to a greater extent than players in the control group.
2. Players in the ACT group will increase the coach rated performance to a greater extent than players in the control group.
3. Players will find ACT meaningful and helpful to them. They will also recommend the training to other players.

## Materials and Methods

### Design and Participants

The present study was initiated and outlined during the season of 2013–2014 as part of the collaboration between Stockholm University and MODO hockey, an elite ice hockey organization in Sweden. The ice hockey players in the organization were invited to participate in the study at a verbal presentation on sport psychology held by the ice hockey organization. The inclusion criteria were: At least 16 years old, male, playing at elite junior level participating in the junior national championships, able and willing to participate in the study. Exclusion criteria was players not eligible to participate in the competition mentioned in the inclusion criteria. The design of the study was a longitudinal RCT with repeated measures at pre, post and at 3-months follow up. Participants were randomized into two groups, either an experimental or a waiting-list control group. Block randomization on individual level with even allocation to both groups was conducted by a blinded independent researcher using a computerized randomization table. In total, 34 participants were included and 16 were randomized to the experimental group (ACT) and 18 to the (waiting list) control group. One participant dropped out of the study before randomization due to long term injury. All participants were males between 16–20 years of age (*M* = 18.09 and SD = 0.88). Participants consisted of 22 forwards and 11 defenders. Of the 34 participants 10 were junior national team players. Position and national team level were matched in randomization. All participants played in the highest junior elite league in Sweden (the junior national championships) and studied at high school level. Study procedures was carried out in accordance with the Declaration of Helsinki (World Medical Association General Assembly, 2004), reviewed and approved by the review committee at the Department of Psychology at Stockholm University, Sweden. All participants received written information about the study and returned a signed informed consent.

### Material

#### Objective Performance

Objective performance measures were collected from the Swedish national hockey association, through the statistical data base swehockey.se. Statistics collected and analyzed was number of games, assists, goals, shots and plus/minus (a statistic where a player who is on the ice when their own team scores get a “plus,” and when the player is on the ice when the opponent team scores get a “minus”; total plusses and minuses are then summed together) through the whole season. Pre-measures were mean scores collected from the start of the season to the start of the intervention, post measures were collected during the training period (mean scores from the start of the intervention and to the end of it), and measures at 3 months follow up were mean scores collected from at the end of the intervention for 3 months. Thus, the objective performance measures were mean statistics from three measurement periods. The statistics were reported into the data base by blinded assessors.

#### Coach Ratings of Player’s Focus, Engagement, and Performance

Coaches rated players, on a seven-point scale, regarding their ability to: (1) focus (*During the last 2 weeks the player has been focusing on the important things during games*); (2) be engaged (*During the last 2 weeks the player has been engaged during practices and games*); and (3) perform (*How good do you think that the player has been in games during the last 2 weeks?*) during games. The seven-point scale for the first two questions ranged from 1 = never true to 7 = always true, and the last question from 1 = always bad to 7 = always good. The scale was discussed at a 3-h seminar between raters and the first author to ensure interrater reliability. The seminar was led by the first author of the study and questions about scale steps was central in the discussions. The raters were the player’s everyday coaches blinded to which group the players were assigned to. Players from the experimental group and the control group were equally divided between the raters. Two coaches rated half the group, and two coaches rated the other half. Intraclass correlation was0.72 and0.79 for the first and the second pair, respectively.

#### Credibility

An adapted version of the credibility questionnaire (Devilly and Borkovec, 2000) was used to evaluate the feasibility of the training program and was assessed at post measure. The items were; (1) Has the intervention been important for you as an ice hockey player, (2) has the intervention helped you to develop as an ice hockey player, and (3) would you recommend the training program to other ice hockey players. Items 1 and 2 were rated on a 1–4 point scale (1 = not at all, 2 = a little bit, 3 = much, 4 = very much) while item 3 was rated either yes or no.

#### Procedure

Players were invited to take part in the study at a presentation on psychology held by the ice hockey organization by the first author. All players who wanted to participate in the study and met the inclusion criteria gave written informed consent. Players were thereafter randomized into either the ACT group or the waiting list by a blinded independent researcher. 16 players were randomized into the experimental group and 18 into the waitlist control. Players were divided into groups of 6–8 players. The training sessions were delivered in a conference room at the ice hockey organization. Self-report measurements were administered directly after the last session. Questionnaires were administered by an independent researcher in the same room as the training took place. Coaches (who also rated the players’ performances) were blinded to which group the players were assigned to. Players in the wait-list control group were given the opportunity to take part of the training after the experiment was completed.

#### Training

An ACT manual adapted for ice hockey players had been developed by the first author (described in more detail: Lundgren et al., 2020). The first author conducted all training. The training program consisted of 4 weekly group sessions between 30–45 min long. There were 6–8 players in each of the two intervention groups that constituted the experimental condition. Before the training started, participants were given a folder with the training material. At the beginning of every session players were handed the new material for the upcoming session. At the end of each session participants were given homework assignments. The psychological trainer called all participants once a week by the phone to answer questions about the material and give feedback on the work progress. Each session followed a similar structure; (1) Introduction of the overall purpose of the training, (2) introduction of the central skill trained in that specific session, (3) an experiential exercise to exemplify the skill and help the players understand and train the specific psychological skill, (4) summary of the session and description of the homework assignment. The training aimed to increase players ice hockey related psychological flexibility. The key processes taught were ice hockey values, acceptance, mindful awareness and committed actions.

### Statistical Analysis

Mixed model repeated measure (MMRM) was used for the repeated measures regarding goals, assist, shots, plus/minus and the coach ratings of focus, engagement and performance. MMRM is an established analytic model, where both fixed and random effects are modeled, for repeated measures in RCTs (Hesser, 2015). Random intercept and random slope were investigated for each outcome model. The repeated statement in SPSS was used to investigate for longitudinal covariance structures. If the random and repeated effects provided better fit they were retained in the model. The default covariance structure, unstructured, compound symmetry, heterogenous compound symmetry, autoregressive, heterogenous autoregressive and Toeplitz were investigated. We included three within assessments (time was coded 0, 4 and 12 modeling the weeks of assessment) and two between levels (ACT and wait-list). The difference in −2 Log likelihood values was used to determine if additional parameters improved the models fit. Quadratic time was included due to non-linear change over time on the dependent variables. Cohen’s *d* was used as effect size estimate. SPSS version 26 was used for all analyses.

## Results

There were no significant differences at preassessment between the groups on the objective measures (0.071 < *p* < 0.495) but the ACT group were rated significantly lower by the blinded coaches compared to the wait-list group (0.006 < *p* < 0.008) on all three variables. Random intercept (not time or squared time) improved all models fit significantly (using the default covariance structure scaled identity). Since random intercept was retained in all models the significant differences on premeasures are accounted for. The effects of autocorrelations did not improve model fit in any outcome model. Due to very small SD’s around the estimated means the effect sizes based on those estimates are large. Due to that effect sizes based on observed means and SD’s are also reported (see Table 1). Differences in N for the coach ratings compared to the objective measures were due to injuries, players being put on the bench (see Table 1).

**TABLE 1:** Estimated means and standard deviations based on the mixed model repeated measures for game statistics and blinded coach ratings from pre to follow up with effect sizes between groups at post and follow up.

	**Pre**		**Post**			**3 month follow up**		
	**ACT *n* = 16 M(SD)**	**WL *n* = 18 M(SD)**	**ACT *n* = 16 M(SD)**	**WL *n* = 18 M(SD)**	**ES *d*^*a*^*d*^*b*^**	**ACT *n* = 16 M(SD)**	**WL *n* = 18 M(SD)**	**ES *d*^*a*^*d*^*b*^**
Goals/game	0.37 (0.10)	0.20 (0.09)	0.50 (0.10)	0.12 (0.09)	4.05 1.46	0.28 (0.10)	0.20 (0.09)	0.78 0.32
Assist/game	0.48 (0.15)	0.33 (0.15)	0.69 (0.15)	0.30 (0.15)	2.58 0.92	0.47 (0.15)	0.23 (0.15)	1.60 1.11
Shots/game	2.08 (0.39)	1.57 (0.45)	2.75 (0.39)	1.58 (0.45)	2.75 1.37	2.07 (0.39)	1.42 (0.45)	1.54 1.04
Plus/minus	0.72 (0.08)	0.60 (0.11)	0.96 (0.08)	0.38 (0.11)	6.06 0.72	0.45 (0.08)	0.31 (0.11)	1.47 0.45
	*n* = 15	*n* = 11	*n* = 15	*n* = 11		*n* = 15	*n* = 11	
Focus	7.47 (0.08)	9.91 (0.05)	10.00 (0.08)	9.18 (0.05)	11.53 0.59	9.60 (0.08)	10.73 (0.05)	−15.89 −0.69
Engagement	7.67 (0.83)	10.18 (0.72)	10.00 (0.83)	10.09 (0.72)	−0.11 −0.05	9.80 (0.83)	11.18 (0.72)	−1.76 −0.81
Performance	7.40 (0.12)	9.45 (0.10)	9.33 (0.12)	9.45 (0.10)	−1.08 −0.07	9.67 (0.12)	10.91 (0.10)	−11.05 −0.72

*WL, wait-list, ES, Cohen’s d between group effect size.*

*^*a*^Between group effect size based on estimated means and SD.*

*^*b*^Between group effect size based on observed means and SD.*

*Due to the estimated means small standard deviations observed means and standard errors around the means are used to present the change within each group for both game statistics and coach ratings.*

### Game Statistics

There were significant interaction effects (group by time and group by quadratic time) for goals per game (see Table 2). The ACT participants showed a non-significant increase from pre- to posttreatment [mean increase = 0.13, *SE* = 0.08, *t*(15) = 1.55, *p* = 0.14, *d* = 0.39] followed by a medium significant decrease from post to follow-up [mean decrease = 0.22, *SE* = 0.10, *t*(15) = 2.24, *p* = 0.041, *d* = 0.56]. The WL participants, on the other hand, showed a decrease from pretreatment to posttreatment [mean decrease = 0.08, *SE* = 0.05, *t*(17) = 1.48, *p* = 0.16, *d* = 0.35] followed by an increase from post to follow-up [mean increase = 0.08, *SE* = 0.06, *t*(17) = 1.35, *p* = 0.19, effect size = 0.32].

**TABLE 2:** Group, time, squared time and interaction effects from the mixed model repeated measures analysis.

	**Group *F*(df)**	**Time *F*(df)**	**Group × time *F*(df)**	**Time^2^*F*(df)**	**Group × time^2^*F*(df)**
Goals/game	4.11(1,89.2)*	0.33(1,68)	4.85(1,68)*	0.8(1,68)	7.36(1,68)**
Assist/game	1.97(1,80.4)	2.74(1,68)	4.31(1,68)*	4.57(1,68)*	4.15(1.68)*
Shots/game	3.42(1,74.5)	5.59(1,66)*	4.94(1,66)*	7.65(1,66)**	5.23(1,66)*
Plus/minus	0.39(1,99.8)	0.06(1,68)	3.32(1,68)	0.85(1,68)	3.92(1,68)
Focus	13.54(1,77.9)***	3.25(1,52)	12.48(1,52)***	1.63(1,52)	12.30(1,52)***
Engagement	12.53(1,65.5)***	6.38(1,52)*	8.43(1,52)**	3.76(1,52)	8.09(1,52)**
Performance	9.92(1,77.8)**	3.76(1,52)	4.61(1,52)*	1.51(1,52)	4.51(1,52)*

*p < 0.05, **p < 0.01, and ***p < 0.001.

There were significant interaction effects (group by time and group by quadratic time) and a significant quadratic time effect for assists per game (see Table 2). The ACT participants showed a non-significant increase from pretreatment to posttreatment [mean increase = 0.21, *SE* = 0.11, *t*(15) = 1.96, *p* = 0.07, *d* = 0.49] followed by a small significant decrease from post to follow-up [mean decrease = 0.22, *SE* = 0.11, *t*(15) = 1.97, *p* = 0.07, *d* = 0.49]. The WL participants showed a decrease from pretreatment to posttreatment [mean decrease = 0.03, *SE* = 0.08, *t*(17) = 0,37, *p* = 0.71, *d* = 0.09] followed by a decrease from post to follow-up [mean decrease = 0.08, *SE* = 0.08, *t*(17) = 0.92, *p* = 0.37, effect size = 0.09].

There were significant interaction effects (group by time and group by quadratic time) and significant time and quadratic time effects for shots per game (see Table 2). The ACT participants showed a significant increase from pretreatment to posttreatment [mean increase = 0.67, *SE* = 0.25, *t*(14) = 2.66, *p* = 0.019, *d* = 0.69] followed by a significant decrease from post to follow-up [mean decrease = 0.67, *SE* = 0.20, *t*(14) = 3.33, *p* = 0.005, *d* = 0.86]. The WL participants showed an increase from pretreatment to posttreatment [mean increase = 0.01, *SE* = 0.20, *t*(17) = 0.05, *p* = 0.96, *d* = 0.01] followed by a decrease from post to follow-up [mean decrease = 0.16, *SE* = 0.21, *t*(17) = 0.76, *p* = 0.46, effect size = 0.18].

There were significant interaction effects (group by time and group by quadratic time) and significant time and quadratic time effects for shots per game (see Table 2). The ACT participants showed a significant increase from pretreatment to posttreatment [mean increase = 0.67, *SE* = 0.25, *t*(14) = 2.66, *p* = 0.019, *d* = 0.69] followed by a significant decrease from post to follow-up [mean decrease = 0.67, *SE* = 0.20, *t*(14) = 3.33, *p* = 0.005, *d* = 0.86]. The WL participants showed an increase from pretreatment to posttreatment [mean increase = 0.01, *SE* = 0.20, *t*(17) = 0.05, *p* = 0.96, *d* = 0.01] followed by a decrease from post to follow-up [mean decrease = 0.16, *SE* = 0.21, *t*(17) = 0.76, *p* = 0.46, effect size = 0.18].

There were no significant effects for plus minus (see Table 2). The ACT participants showed a significant increase from pretreatment to posttreatment [mean increase = 0.24, *SE* = 0.19, *t*(15) = 1.24, *p* = 0.23, *d* = 0.31] followed by a significant decrease from post to follow-up [mean decrease = 0.51, *SE* = 0.24, *t*(15) = 2.16, *p* = 0.047, *d* = 0.54]. The WL participants showed a decrease from pretreatment to posttreatment [mean decrease = 0.22, *SE* = 0.16, *t*(17) = 1.35, *p* = 0.195, *d* = 0.32] followed by a decrease from post to follow-up [mean decrease = 0.07, *SE* = 0.23, *t*(17) = 0.31, *p* = 0.76, effect size = 0.07].

### Coach Ratings

There were significant interaction effects (group by time and group by quadratic time) and significant group effects for ratings of players Focus (see Table 2). The ACT participants showed a significant increase from pretreatment to posttreatment [mean increase = 2.53, *SE* = 0.78, *t*(14) = 3.25, *p* = 0.006, *d* = 0.84] followed by a non-significant decrease from post to follow-up [mean decrease = 0.40, *SE* = 0.48, *t*(14) = 0.84, *p* = 0.415, *d* = 0.22]. The WL participants showed a decrease from pretreatment to posttreatment [mean increase = 0.73, *SE* = 0.70, *t*(10) = 1.04, *p* = 0.32, *d* = 0.31] followed by a significant increase from post to follow-up [mean increase = 1.55, *SE* = 0.62, *t*(10) = 2.48, *p* = 0.033, *d* = 0.75].

There were significant interaction effects (group by time and group by quadratic time), significant group and time effects for ratings of players Engagement (see Table 2). The ACT participants showed a significant increase from pretreatment to posttreatment [mean increase = 2.33, *SE* = 0.77, *t*(14) = 3.04, *p* = 0.009, *d* = 0.79] followed by a non-significant decrease from post to follow-up [mean decrease = 0.20, *SE* = 0.35, *t*(14) = 0.56, *p* = 0.58, *d* = 0.14]. The WL participants showed a decrease from pretreatment to posttreatment [mean decrease = 0.09, *SE* = 0.25, *t*(10) = 0.16, *p* = 0.88, *d* = 0.05] followed by a significant increase from post to follow-up [mean increase = 1.09, *SE* = 0.25, *t*(10) = 4.35, *p* = 0.001, *d* = 1.31].

There were significant interaction effects (group by time and group by quadratic time) and a significant group effect for ratings of players Performance (see Table 2). The ACT participants showed a significant increase from pretreatment to posttreatment [mean increase = 1.93, *SE* = 0.75, *t*(14) = 2.57, *p* = 0.022, *d* = 0.66] followed by a non-significant increase from post to follow-up [mean increase = 0.33, *SE* = 0.57, *t*(14) = 0.59, *p* = 0.56, *d* = 0.15]. The WL participants showed no change from pretreatment to posttreatment [mean change = 0, *SE* = 0.50, *t*(10) = 0, *p* = 1, *d* = 0] followed by a significant increase from post to follow-up [mean increase = 1.45, *SE* = 0.51, *t*(10) = 2.85, *p* = 0.017, effect size = 0.86].

The credibility/feasibility ratings from the ACT group (n = 16) were as follows:

(1) Has the intervention been important for you as an ice hockey player? 44% rated very much, 50% rated much and 6% a little bit. (2) Has the intervention helped you to develop as an ice hockey player? 22% rated very much, 22% much and 56% a little. Question (3) would you recommend the training program to other ice hockey players? 100% of the players stated yes.

## Discussion

The results of the study show that a brief group intervention with Acceptance and Commitment Training (ACT) significantly increases performance on measures such as goals, assists and shots as compared to a wait-list control. Using blinded coach ratings, players in the ACT group received a superior increase in ratings of performance, being focused on important aspects of their ice-hockey and being engaged in their development in comparison to the wait list control. Also, the ice hockey players in this study considers the ACT training to be meaningful and helpful in their development as ice hockey players and 100% would recommend the training to other ice hockey players. To our knowledge this is the first study using a randomized controlled group design to evaluate the effect of an acceptance, mindfulness and values-based intervention on objective performance measures in ice hockey. No long-term effects were found on objective performance following this 4-session training which suggests further research is needed that also focus on the maintenance of enhanced skills. However, results on blinded coach ratings lasted at 3 months follow up.

Acceptance and Commitment Training is a behavioral change method well-suited to adapt to a range of varying contexts since it constitutes of general behavioral change processes (Hayes et al., 1999). The feasibility of the current ACT group intervention have been tested with satisfying results (Lundgren et al., 2020), and the results of this study further add to the growing body of evidence for using acceptance-based behavioral interventions in sports. Notably for its effectiveness as a performance enhancement method in ice hockey. As mentioned previously, several systematic reviews in the field have concluded that studies using a high-quality methodology such as an RCT design is needed (Sappington and Longshore, 2015; Bühlmayer et al., 2017; Noetel et al., 2019), which this study is an addition to.

Performance measurements in team sports in general, and ice hockey more specifically, need to be addressed in relation to the present study. Even though participants were randomized, the success of an individual in a team is dependent on his or her teammates. In the present study for example, number of scored goals by an individual are dependent upon teammates performances (e.g., creating space, assists and goal-scoring opportunities). Furthermore, a successful assist-maker needs someone to finish it off and score the goal. It is common in sport psychology to investigate intervention effects on performance surrogates and psychological processes deemed important for the intervention and athletic performance (Bühlmayer et al., 2017). Such focus in outcomes is also very important and may lead to valuable findings, however, it answers different types and dimensions of research questions. As such, if change in performance itself is part of the aim for the psychological training—then direct performance should be measured in some way. With that said, both objective and subjective procedures to measure performance have its risks of being flawed or insensitive in different ways (Weinberg, 1990; Raglin, 1992). As mentioned above regarding objective performance parameters, there is a risk of them being altered by several other external factors not related to the experiment. Also, using an objective sport specific outcome measure does not automatically imply that a high-quality outcome has been adopted. Ultimately, the quality of a performance outcome measure is highly dependent on reflecting desired performance behavior. This is a critical validity issue in sport psychology measurement overall. In the present study, both objective performance measures and blinded coach ratings were used as outcome in order to capture different aspects and perspectives of performance, since no single way of measuring is ideal as mentioned above. Direct effects on ice hockey performance such as goals, assists, shots taken are important results in a result focused world such as competitive sports, and specifically for this study in ice hockey to gain acknowledgment in the ice hockey world. Addressing direct effects add important knowledge. The combination between studying direct effects and mediators such as flow, mindfulness, acceptance or other processes of interest is an important aim for future research.

The results in this study would have been strengthened with an even larger sample size and also if the players would have been included from more than one ice hockey organization. This would have been favorable for the generalization of the results from the study. In future research, larger group trials will also help to further bottom out possible performance level/outcome differences between experimental conditions at baseline, even if randomization and appropriate matching procedures are being used. As in this study, randomization procedures were used, and players were also matched based on national team participation. However, the experimental group tended to have higher coach rating values at baseline compared to the control group, and a reversed tendency could be seen for some of the objective outcome measures (Table 2). With larger samples size such within-sample variations are more likely to even out in a desirable way. Although statistical procedures can take this into account on a statistical level, larger sample sizes minimize the risk of actual performance differences at baseline that otherwise unknowingly may exist.

Since the ACT training targets PF skills such as acceptance, mindfulness and values for athletes, a limitation for this study is that no PF related measure was included. The VAMS scale that measures PF in ice hockey (Lundgren et al., 2018) would have added valuable information to the experiment. Not only are scores on the VAMS positively associated with objective ice hockey performance parameters and aspects of mental health it would also have given the opportunity to investigate whether PF was increased by the ACT program in this study, which was found in a previous trial (Lundgren et al., 2020). Also, the relationship between a potential change in PF and the observed performance increase found in this trial for the ACT group could have been investigated. Another limitation in the current study is that no active comparison or no placebo control condition was used. This also aggravate the interpretations of the improvements in the ACT group, and to what extent the observable effect can be attributed to the experimental manipulation and not due to e.g., potential placebo effects in the intervention group. In future research, it is recommended to include both psychological measures of interest for the intervention as well as performance outcome. Further, an active comparator or a placebo condition would strengthen the research design.

The result in the present study shows that there are no longer any differences between the training group and the control group regarding the objective performance measures at 3-months follow up. In future research, an extended ACT training program could be one aspect to explore how to maintain effects. Since the intervention consisted of only 4 group sessions (á 40 min) plus homework exercises, it may have been on the expense of lasting behavior change and that more training had been needed for the new skills to be fully implemented. Another explanation could be that the training content should have put even more emphasis on the maintenance aspect of the new behavior skills set during the training, which is a crucial part of psychological interventions in general (Barlow, 2014). Elite ice hockey players follow the schedule set by the team staff: physical training, on-ice practices, tactical briefings etc. And while the ACT training was ongoing the sessions were included in the team schedule and players were encouraged to practice the psychological skills as well. However, when the training ended, there were no more prompts for the players to further engage in the ACT training. This also elucidates the role of the sport organization and that when new training procedures are implemented, the organizational structure need to reinforce engagement for the procedure to have long-term effects. Therefore, it would be interesting to explore the effect of longer continuous ACT training stimulated by organizational structures. The inclusions of continuous follow ups and guidance not only on physical training but psychological training as well may increase the likelihood of lasting behavior change.

### Future Research

In order for the effects of ACT interventions in sports to further being established, the multifaced question of generalizability is crucial. First of all, larger group trials with a robust methodology are needed to validate estimated effects. This applies both to the specific sport of ice hockey that was studied in this article as well as a range of other sports. The demands and challenges of being a top athlete and the central role of psychological functioning are non-disputed. However, psychological support and training can be tailored differently depending on matters such as type of sport to make the intervention more sport-specific (Josefsson et al., 2020), what challenges the athlete encounter, and in what phase the athlete is in his or her sporting career. In the growing empirical base of contextual behavioral approaches in sport, such differences in target populations, intervention content and formats need to be further investigated.

In order to further research how ACT training can be of help for athletes, new formats of delivering interventions may also be of interest to investigate. In clinical psychology for example, internet-based psychological interventions have proven successful in treating several psychiatric conditions. Moreover, the internet format may also offer treatment to patients that otherwise would not have received a psychological treatment, while also being a cost-effective alternative (Andersson et al., 2019). If internet-based psychological interventions would be evaluated for athletes, this would possibly be an alternative to facilitate the access of psychological services for more athletes overall. Also, since many athletes travels a lot, especially on elite level, a portable internet format could possibly be an ideal benefit for some athlete populations. Another possible advantage of the internet-based format, related to the results of this trial, is how to stimulate continuous psychological skills practice over time to maintain intervention effects. The internet-based format may perhaps at least facilitate that continuous access to further training over time to maintain learned skills.

### Conclusion

The present study shows that a brief ACT program has significant effects on ice hockey players’ objective performance such as goals, assists and taken shots. Further, coaches’ ratings of player performance, focus and commitment to their ice hockey increased for the ACT trained players. The ice hockey players themselves considered the training to be meaningful and helpful to their ice hockey development. This study adds to the growing body of evidence for using ACT with athletes to enhance athletic performance. The results also suggest that ACT could be a valuable addition to ice hockey organizations who wants to take the psychological aspect of ice hockey into account when developing a successful organization. However, more research is needed, especially to explore additional interventions to establish long term effects of the training.

## Data Availability Statement

The raw data supporting the conclusions of this article will be made available by the authors, without undue reservation.

## Ethics Statement

Ethical review and approval was not required for the study on human participants in accordance with the local legislation and institutional requirements. The patients/participants provided their written informed consent to participate in this study.

## Author Contributions

TL organized and designed the study, delivered the training, analyzed data, and wrote the manuscript. GR analyzed data and cowrote the manuscript. MF co-planned the study, analyzed data, and wrote the manuscript. EJ co-planned the study, acted as co-trainer, analyzed data, and wrote the manuscript. MN, US, and PS co-organized the study, developed the training material, and wrote the manuscript. TP co-organized the study, analyzed data, and wrote the manuscript. All authors contributed to the article and approved the submitted version.

## Conflict of Interest

MN, PS and US were employed by the company MoDo Hockey. The remaining authors declare that the research was conducted in the absence of any commercial or financial relationships that could be construed as a potential conflict of interest.
